# Examining the Overlap between Autism Spectrum Disorder and 22q11.2 Deletion Syndrome

**DOI:** 10.3390/ijms18051071

**Published:** 2017-05-18

**Authors:** Opal Ousley, A. Nichole Evans, Samuel Fernandez-Carriba, Erica L. Smearman, Kimberly Rockers, Michael J. Morrier, David W. Evans, Karlene Coleman, Joseph Cubells

**Affiliations:** 1 Emory Autism Center, Department of Psychiatry and Behavioral Sciences, Emory University School of Medicine, 1551 Shoup Court, Atlanta, GA 30322, USA; kimberly.rockers@gmail.com (K.R.); mmorrier@emory.edu (M.J.M.); jcubell@emory.edu (J.C.); 2 Marcus Autism Center, Children’s Healthcare of Atlanta, 1920 Briarcliff Road, Atlanta, GA 30329, USA; andrea.evans@choa.org (A.N.E.); samuel.fernandez-carriba@emory.edu (S.F.-C.); karlene.coleman@choa.org (K.C.); 3 Marcus Autism Center, Department of Pediatrics, Emory University School of Medicine, 1920 Briarcliff Road, Atlanta, GA 30329, USA; 4 Department of Psychology, Emory University, Psychology and Interdisciplinary Studies (PAIS) Building, 36 Eagle Row, Atlanta, GA 30322, USA; esmearm@emory.edu; 5 Department of Human Genetics, Emory University School of Medicine, 615 Michael Street, Whitehead Biomedical Research Building, Suite 301, Atlanta, GA 30322, USA; 6 Department of Psychology, Bucknell University, 1 Dent Drive, Lewisburg, PA 17837, USA; dwevans@bucknell.edu

**Keywords:** 22q11.2 deletion, autism, autism spectrum, diagnosis, copy number variation, CNV, Research Domain Criteria, RDoC

## Abstract

22q11.2 deletion syndrome (22q11.2DS) is a genomic disorder reported to associate with autism spectrum disorders (ASDs) in 15–50% of cases; however, others suggest that individuals with 22q11.2DS present psychiatric or behavioral features associated with ASDs, but do not meet full criteria for ASD diagnoses. Such wide variability in findings may arise in part due to methodological differences across studies. Our study sought to determine whether individuals with 22q11.2DS meet strict ASD diagnostic criteria using research-based guidelines from the Collaborative Programs of Excellence in Autism (CPEA), which required a gathering of information from three sources: the Autism Diagnostic Interview-Revised (ADI-R), the Autism Diagnostic Observational Schedule (ADOS), and a clinician’s best-estimate diagnosis. Our study examined a cohort of children, adolescents, and young adults (*n* = 56) with 22q11.2DS, who were ascertained irrespective of parents’ behavioral or developmental concerns, and found that 17.9% (*n* = 10) of the participants met CPEA criteria for an ASD diagnosis, and that a majority showed some level of social-communication impairment or the presence of repetitive behaviors. We conclude that strictly defined ASDs occur in a substantial proportion of individuals with 22q11.2DS, and recommend that all individuals with 22q11.2DS be screened for ASDs during early childhood.

## 1. Introduction

22q11.2 deletion syndrome (22q11.2DS) is among a growing number of genomic disorders that associate with autism spectrum disorders (ASDs) [1]. 22q11.2DS, also referred to as DiGeorge syndrome, or velo-cardio-facial syndrome (VCFS), occurs in approximately 1/4000 live births [2,3,4,5], making it the most common recurrent copy-number variant (CNV) associated with developmental disorders described to date. 22q11.2DS arises from an interstitial deletion of up to 3 Mb of DNA on chromosome 22q11.2 and produces a diverse range of physical, behavioral, social, and neurocognitive impairments [2,5,6,7], which overlap with the social-communication impairments found among patients with ASD [7,8,9].

While previous studies have described both the presence of ASDs among individuals with 22q11.2DS and the presence of 22q11.2DS among individuals diagnosed with ASD, there is no consensus on the rate of co-occurrence [10]. Previous studies have reported that 15–50% of those with 22q11.2DS also have an ASD [8,9,11], and that between 0.3% and 1% of individuals with an ASD also have 22q11.2DS [12,13]—an approximately 10–40-fold increase relative to the general population. The substantial discrepancies in reported rates of ASDs in 22q11.2DS could reflect methodological differences across studies, including the use of varied subject ascertainment methods and diagnostic assessment approaches [8]. However, some researchers argue that, although ASD-like symptoms are present in individuals with 22q11.2DS, these individuals do not truly meet criteria for an ASD, and may represent misclassified persons with, for example, prodromal symptoms of schizophrenia [14,15].

This issue resembles the ongoing debate faced by Fragile X syndrome (FXS) researchers, on the association between ASD and FXS [16,17,18]. Although debate over the overlap between FXS and autism continues, the clinical evaluation of individuals with ASD now regularly includes molecular testing for FXS. Furthermore, basic research on FXS has led to the testing of novel psychopharmacological interventions for FXS that appear to impact ASD-related symptoms [19,20]. Thus, the value of understanding relationships among molecularly defined genetic and genomic disorders and ASD is abundantly clear.

Our study aims to clarify the association between 22q11.2DS and ASD by applying rigorous, research-based diagnostic methods applied to a cohort of individuals ascertained solely on the basis of a molecular diagnosis of 22q11.2DS. We sought to determine the proportion of individuals with 22q11.2DS who meet research criteria for an ASD diagnosis according to the guidelines set forth by the Collaborative Programs of Excellence in Autism (CPEA) Simons Simplex Collection study (Simons Foundation Autism Research Initiative) [21]. The CPEA guidelines require individuals to meet cutoff scores on gold standard observational and parent report measures (i.e., the Autism Diagnostic Interview-Revised (ADI-R) [22] and Autism Diagnostic Observation Schedule (ADOS) [23]) in conjunction with clinicians’ best-estimate consensus diagnosis in order to receive a research-based diagnosis of ASD. We assessed 56 children, adolescents, and young adults with fluorescent in situ hybridization (FISH) confirmed 22q11.2DS, ascertained from a registry of all cases of 22q11.2DS diagnosed at Children’s Healthcare of Atlanta since the mid-1990s. We hypothesized that we would identify strictly defined ASDs in a substantial proportion of patients with 22q11.2DS.

## 2. Results

**Figure 1:**
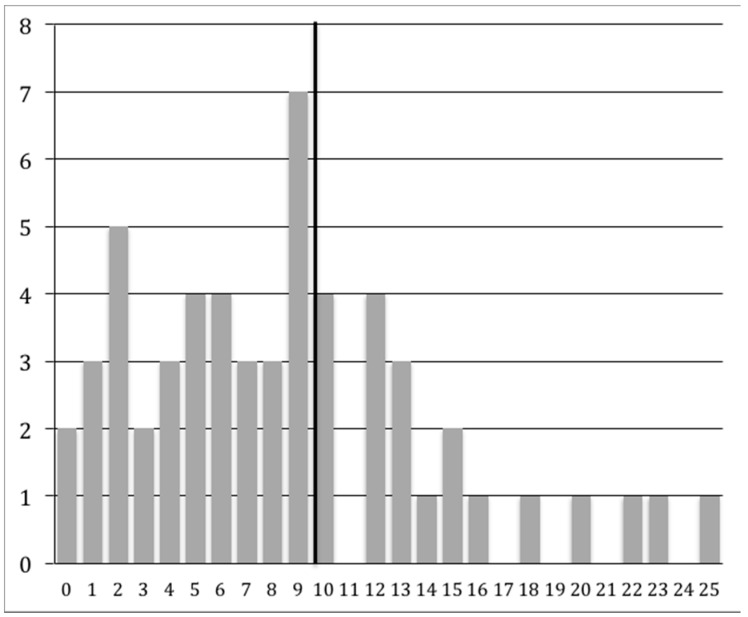
Figure 1 shows the broad range of Autism Diagnostic Interview-Revised (ADI-R) Reciprocal Social Interaction algorithm total scores (0–25; the autism spectrum disorder (ASD) cutoff score ≥ 10 is indicated by the black vertical line). The *x*-axis shows the algorithm score, and the *y*-axis indicates the number of individuals with each score.

**Figure 2:**
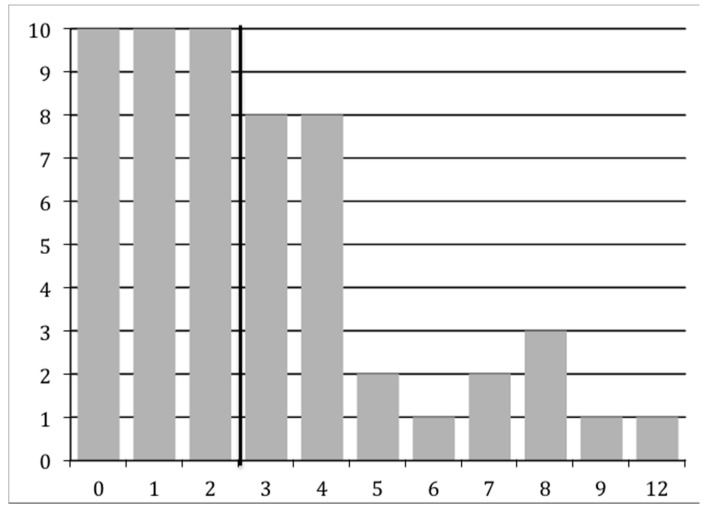
Figure 2 shows the broad range of ADI-R Restricted, Repetitive, and Stereotyped Behavior algorithm total scores (0–12; the ASD cutoff score ≥3 is indicated by the black vertical line). The *x*-axis shows the algorithm score, and the *y*-axis indicates the number of individuals with each score.

**Figure 3:**
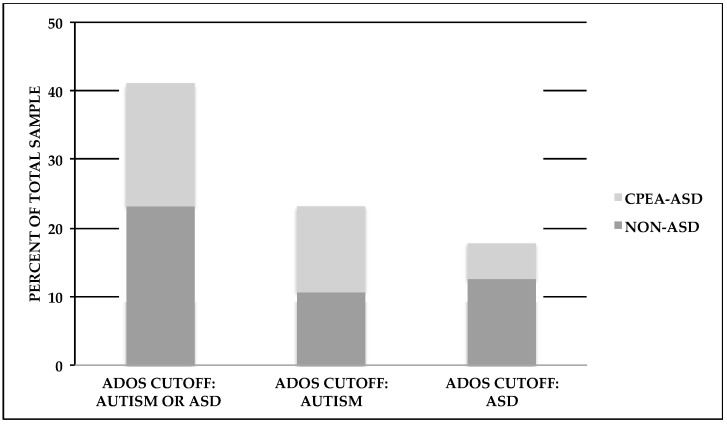
Figure 3 shows the percentages of individuals meeting the Autism Diagnostic Observational Schedule (ADOS) cutoff criteria. Within each cutoff category, diagnostic groups are designated as those who either met the Collaborative Programs of Excellence in Autism (CPEA) criteria for an ASD diagnosis (CPEA-ASD), or those who did not meet these criteria (non-ASD).

**Figure 4:**
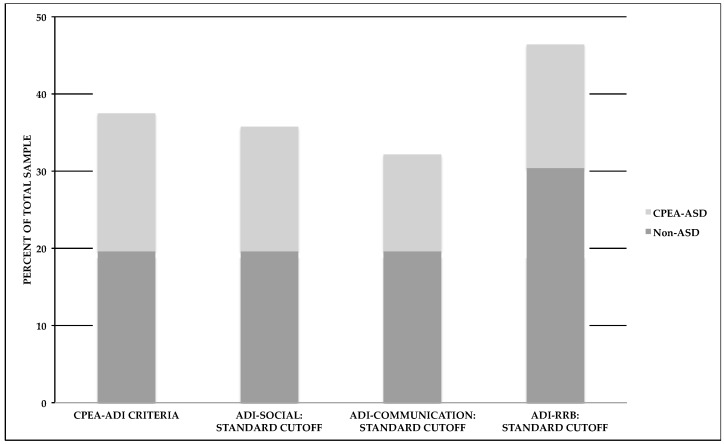
Figure 4 shows the percentages of individuals meeting ADI-R criteria set forth by the CPEA (CPEA-ADI) or standard ADI-R domain cutoff scores. Within each cutoff category, diagnostic groups are designated as those who met CPEA-ASD criteria or those who did not meet these criteria (non-ASD).

We found that a substantial number of individuals with 22q11.2DS within our sample met strictly defined ASD (17.9%, *n* = 10; 2.3:1 male to female ratio), using rigorous research diagnostic criteria, and that a significant level of ASD-related symptoms occurred for a portion of our sample, even in the absence of an ASD diagnosis. An additional subset of individuals did not meet overall autism or ASD cutoffs on the standardized assessments. Details of these findings are outlined below and depicted in Figure 1, Figure 2, Figure 3 and Figure 4.

As described in the Materials and Methods section, we examined various cutoff scores for the Autism Diagnostic Observation Schedule (ADOS) and the Autism Diagnostic Interview-Revised (ADI-R), and determined who met CPEA criteria for an ASD diagnosis. A majority of individuals (*n* = 53) completed either ADOS Modules 3 or 4, which is given to children or adults, respectively, with language fluency. Three individuals completed either Module 1 (*n* = 2) or Module 2 (*n* = 1), which is given to individuals with no speech/single words or phrase speech, respectively. Examination of the ADOS scores revealed that 23.2% (*n* = 13) of the participants met the autism cutoff score and 17.9% (*n* = 10) met the ASD cutoff score, for a total of 41.1% (*n* = 23). We also found that more than a third of the study participants (37.5%, *n* = 21) met ADI-R criteria as specified by the CPEA criteria, which follows either the standard ADI-R cutoff scoring parameters for autism (17.9%, *n* = 10) or a more flexible set of parameters (19.6%, *n* = 11; following Risi et al.; see Methods section below) [24]. An examination of each ADI-R domain score revealed that 35.7% (*n* = 20) met the ADI-R Reciprocal Social Interaction domain cutoff score, 32.1% (*n* = 18) met the ADI-R Communication domain cutoff score, and 46.4% (*n* = 26) met the Restricted, Repetitive, and Stereotyped Behavior domain cutoff score. Moreover, 66.1% (*n* = 37) of our total sample met cutoff scores for at least one of the social, communication, or repetitive behavior domains on the ADI-R. A wide range of algorithm scores was obtained for the ADI-R: Reciprocal Social Interaction range = 0–25, Communication range (verbal algorithm) = 0–18 (*n* = 53) and Communication range (nonverbal algorithm) = 0–12 (*n* = 3), and Restricted, Repetitive, and Stereotyped Behavior range = 0–12. The range of scores for the Reciprocal Social Interaction algorithm is shown below in Figure 1, whereas the range for the Restricted, Repetitive, and Stereotyped Behavior algorithm score is shown in Figure 2.

We also examined the presence of a strictly defined ASD using the CPEA guidelines, which required us to examine cutoff scores on the both the ADI-R and ADOS, in conjunction with best-estimate Diagnostic and Statistical Manual of Mental Disorders, Fourth Edition (DSM-IV) diagnosis [21,25]. We found that, of 56 participants, 17.9% (*n* = 10) met CPEA research-based criteria for a strictly defined ASD. That is, each participant met cutoff criteria for both the ADI-R and ADOS, and received a best-estimate diagnosis of a DSM-IV ASD. Our participants meeting CPEA criteria included individuals with a best-estimate diagnosis of DSM-IV Autistic Disorder or PDD-NOS. Although DSM-IV Asperger’s Disorder would have qualified as a CPEA diagnosis, no participants in this sample met Asperger’s diagnostic criteria. For those individuals who did not meet CPEA diagnostic criteria, two subsets emerged. In one subset (39.3%, *n* = 22), neither the ADI-R nor the ADOS overall cutoff scores were met; in the second subset (42.9%, *n* = 24), overall cutoff scores were met for at least one of the standardized instruments. Thus, even in the absence of a CPEA-ASD diagnosis, a significant minority of individuals met ADOS cutoff scores, or ADI-R cutoff scores, depicted in Figure 3 and Figure 4.

## 3. Discussion

Debate continues regarding the association between 22q11.2DS and ASDs. While multiple studies show that individuals with 22q11.2DS present with ASD-like symptomatology, not all studies find that such individuals qualify for a formal diagnosis of an ASD [26]. Our study attempts to resolve these discrepant findings by considering ascertainment bias and drawing from a strict and thorough research-based definition of ASD, as outlined by CPEA guidelines, which require individuals to attain cutoff scores for an ASD from both the ADI-R and ADOS as well as a clinician’s best estimate diagnosis of DSM-IV Autism, Asperger’s Disorder, or PDD-NOS. Our findings showed that a substantial proportion of study participants with 22q11.2DS met research-based CPEA criteria for an ASD, supporting an association between 22q11.2DS and ASD. We also found in our sample that significant social and communication impairments, as well as restricted and repetitive behaviors, occurred in the absence of an ASD diagnosis.

Our finding that 17.9% of study participants with 22q11.2DS met research CPEA criteria for an ASD diagnosis falls within the range of prior studies reporting that 15–50% of individuals meet diagnostic criteria for an ASD [8,9,11]. To obtain a conservative estimate of ASD in individuals with 22q11.2DS, we chose (1) to use the CPEA research criteria; and (2) to recruit from a hospital and medical clinic database of individuals with 22q11.2DS, with a molecular diagnosis of the disorder as the only criterion for participation. We thus avoided the potential ascertainment bias inherent in studying subjects independently seeking behavioral, mental health, or neurodevelopmental evaluations. Importantly, while this is not an epidemiologic study and individuals in this sample may not be representative of all individuals with 22q11.2DS, a substantial portion of participants met ASD criteria, suggesting the presence of an association. We also found that almost 40% of 22q11.2DS participants met neither the ADI-R nor the ADOS overall cutoffs for autism or ASD, but that more than 60% met at least one domain cutoff on the ADI-R.

These findings should be considered in light of the RDoC (Research Domain Criteria) initiative of the National Institutes of Mental Health [27]. Here, we applied a conservative threshold for ASD diagnosis. We recognize, however, that neurodevelopmental disorders are characterized by deviations and delays on dimensional traits that extend well into the general population [28,29]. Thus, understanding the morbidity of CNV syndromes requires an appreciation for the more subtle deviations and delays that may arise throughout development, reflecting, for example, a psychiatric prodrome. In some instances, even probands with de novo mutations that appear clinically unaffected nonetheless exhibit a “shift” in symptom expression relative to non-carrier first-degree relatives [30,31]. It is important therefore to bear in mind the impact of a CNV as it increases risk—both in terms of traditional clinical thresholds and categories, as well as dimensions of cognitive, social, motor, and behavioral phenotypes.

Our study’s limitations include its cross-sectional design, which precludes examination of behavioral trajectories or emergence of ASD-related symptoms. Additionally, although the presence of co-morbid diagnoses was not the focus of this paper, individuals with 22q11.2DS are at risk for symptoms that extend beyond an ASD diagnosis, including schizophrenia spectrum disorder, depressive disorders, and anxiety disorders [21,32]. We do not yet know precisely how the presence of an ASD diagnosis or ASD symptoms in the absence of an ASD diagnosis affects the emergence of these additional disorders, and only longitudinal studies will allow us to understand these associations. Further, our study relied on the DSM-IV diagnostic system, which closely mirrors the International Classification of Diseases (ICD-10) published by the World Health Organization; however, we did not re-code our autism diagnoses based on the revised specifications for “autism spectrum disorder” outlined in the Diagnostic and Statistical Manual, Fifth Edition (DSM-5). We would predict that our findings would be similar using the DSM-5 criteria, which were developed in part based on prior research from the ADOS, ADI-R, and CPEA [21,23,24]. Future studies examining the various diagnostic classification systems are needed, however, to verify the stability of our findings.

Findings from this study have both clinical and scientific relevance. Because this study supports an association between 22q11.2DS and ASD, we recommend that individuals with 22q11.2DS (1) receive earlier evaluations for ASDs and (2) receive targeted interventions and therapies, such as pharmacological and behavioral approaches, which, when started earlier, may lead to more positive long-term outcomes [9,19]. Furthermore, clinicians evaluating or treating patients with ASDs should consider diagnostic testing for 22q11.2DS (and other CNV disorders), particularly when other associated features such as congenital heart defects, velo-palatal pathology, and immune-related difficulties are present. Awareness of the association between 22q11.2DS and ASDs may also lead to specific hypotheses regarding the etiology and final common pathways that increase risk for childhood social disability and ASDs [33,34].

## 4. Materials and Methods

### 4.1. Participants

#### 4.1.1. Overview

This study evaluated 56 participants between the ages of 6–29 who were diagnosed with 22q11.2DS and who had participated in a larger study investigating neuropsychological and behavioral outcomes in 22q11.2DS [21,35]. All individuals in the study underwent neurocognitive screening and diagnostic assessments for ASD, which included the Autism Diagnostic Interview- Revised (ADI-R) [22] and the Autism Diagnostic Observation Schedule (ADOS) [23]. Data collection procedures were approved by the Emory University Institutional Review Board (#IRB00024756). 

#### 4.1.2. Recruitment and Eligibility

We recruited study participants from a children’s 22q11.2DS medical clinic and hospital case registry maintained at the Children’s Healthcare of Atlanta, which also includes adult subjects who were first diagnosed with 22q11.2DS in a congenital heart defects follow-up clinic. These clinics serve all of metro Atlanta and pull from a large regional catchment area in the Southeastern United States. For all study participants, the diagnosis of 22q11.2DS was identified by FISH using the standard *TUPLE* probe (LSI TUPLE1(22q11.2)/ARSA(22q13.3); Abbott Molecular #05J21-028; Abbott Park, IL, USA). This probe detects the common 3 Mb and 1.5 Mb deletions, which account for approximately 90% of the deletions observed in the clinically ascertained in the DiGeorge and velo-cardio-facial syndrome populations, but does not distinguish between these deletions. 

Having a diagnosis of 22q11.2DS was the primary eligibility requirement for each study, and recruitment occurred without regard to the level of behavioral, developmental, or medical difficulties. The adolescent/adult participants were recruited for a longitudinal study of psychiatric symptoms in 22q11.2DS, and the child participants were recruited for a longitudinal study investigating language development. A majority of subjects (*n* = 51) were ascertained directly from this case registry, based on age eligibility. Five other participants were referred directly to the study from the Children’s Healthcare of Atlanta 22q11.2DS multidisciplinary medical clinic or an adult congenital heart clinic, which follows children formerly served at Children’s Healthcare of Atlanta. Additionally, no subjects in the study were from the same family.

#### 4.1.3. Demographic Characteristics

Our 56 participants include an adolescent/adult group (*n* = 32; mean age = 19.21, SD = 4.12; range = 14–29) and a child group (*n* = 24; mean age = 9.58, SD = 1.91; range = 6–11). For the adult group, females comprise 53.13% (*n* = 17) of the group, while among children, females comprise 45.83% (*n* = 11). The majority of our adult participants are Caucasian (78.12%, *n* = 25) with three additional ethnicities represented: African-American (12.50%, *n* = 4), Hispanic (6.25%, *n* = 2), and Asian (3.12%, *n* = 1). Similarly among our child participants, Caucasian (70.83%, *n* = 17) is the majority, with four additional ethnicities represented: Hispanic (12.50%, *n* = 3), African-American (8.33%, *n* = 2), Asian (4.16%, *n* = 1), and bi-racial: African-American and Caucasian (4.16%, *n* = 1). Parent-reported medical, developmental and family history is provided in Table 1, which indicates a high rate of cardiac, immune, and palatal problems in the study participants. Parents also commonly reported a history of speech delay and ADHD in the study participants, although a history of known autism spectrum disorder was reported for only one participant. Parents reported ADHD as the most common disorder among family members (i.e., parents or siblings); only one family member was known to have 22q11.2DS.

For the adolescent/adult group, the estimated Verbal IQ (VIQ) was based on the Vocabulary and Similarities subtests from either the Wechsler Adult Intelligence Scale–Third Edition (WAIS-III) or the Wechsler Intelligence Scale for Children-Third Edition (WISC-III), depending on chronological age. To calculate the estimated VIQ, we converted the T-scores from these subtests to standard scores, and calculated their average (i.e., mean VIQ = 84.61, SD = 16.81; range = 57–120; *n* = 32). We also converted the WAIS-III or WISC-III Block Design T-score to a standard score to determine an estimated nonverbal IQ (NVIQ) (i.e., mean NVIQ = 73.29, SD = 14.96; range = 55–100; *n* = 32). For the child group, we used the Differential Ability Scales–Second Edition (DAS-II) Verbal score as the indicator of VIQ (i.e., mean VIQ = 72.87; SD = 17.60; range = 3–98; *n* = 23) and used the DAS-II Pattern Construction score to estimate NVIQ (i.e., mean NVIQ = 79.48, SD = 15.69; range = 43–102; *n* = 22). The sample sizes used to calculate the children's VIQ and NVIQ mean scores vary by one and two data points, respectively, due to missing data.

### 4.2. Diagnostic Procedures

We applied a strict, research-based definition of ASD, as designated by CPEA guidelines and outlined by the Simons Simplex Collection study (Simons Foundation Autism Research Initiative), previously described [21]. Essentially, these criteria require participants to attain cutoff scores for an autism spectrum disorder from all of the following sources: (1) the Autism Diagnostic Interview-Revised (ADI-R); (2) the Autism Diagnostic Observation Schedule (ADOS); and (3) the clinician’s best estimate DSM-IV diagnosis, which considers a hierarchical classification strategy for identifying autism, Asperger’s Disorder, or PDD-NOS [25]. To achieve the clinician’s best estimate diagnosis, two clinicians conducted a case conference during which they reviewed all diagnostic and neurocognitive assessments available.

### 4.3. Diagnostic Assessments

The diagnostic assessments included the Autism Diagnostic Interview-Revised (ADI-R) and the Autism Diagnostic Observation Schedule (ADOS) [22,23]. The ADI-R is a semi-structured interview used to evaluate autistic symptomatology and, in particular, to differentiate between autism and other developmental disorders [22]. It is used for a wide range of ages, with a minimum mental age of 18 months. As such, both adults and children were assessed with the ADI-R. This interview evaluates three functional domains: Reciprocal Social Interaction, Communication skills, and Restricted, Repetitive, and Stereotyped behaviors. We classified participants as meeting CPEA-ADI-R cutoff criteria if they met either the standard ADI-R cutoff scores, or an additional set of cutoff scores suggested by Risi et al. [24]. The standard cutoff scores included (1) the total Social algorithm score ≥10; (2) the total (verbal) Communication algorithm score ≥8 or the total (nonverbal) Communication algorithm score ≥7; and (3) the total Restricted, Repetitive and Stereotyped behaviors algorithm score ≥3 [22]. ADI-R cutoff rule, as designated by the Collaborative Programs of Excellence in Autism (CPEA), required that one of the following conditions be met: (1) meeting standard cutoffs for both Social and Communication domains; (2) meeting the standard cutoff for the Social domain and within 2 points of the standard Communication domain cutoff; (3) meeting the standard cutoff for Communication domain and within 2 points of standard Social domain cutoff; or (4) meeting the standard cutoff within 1 point for both the Social and Communication domains [24].

The Autism Diagnostic Observation Schedule (ADOS) is a semi-structured assessment that uses tasks and play activities to elicit social communication and interaction [23,36]. Observations are made concerning social and communication behaviors associated with ASD. Its structure allows for diagnoses across all ages, developmental stages, and language abilities and includes four different modules (1–4) dependent on age, development, and language ability [36]. We used ADOS for both adults and children. For this study, we used the revised algorithms for Modules 1, 2, and 3 were used based on Gotham et al. [23], whereas the standard algorithm for Module 4 was used. Please note, although we used the first edition of the ADOS in combination with the revised algorithms, these revised algorithms are parallel to the cutoffs used for the second revision of the ADOS (i.e., ADOS-2).

Modules 1–3 consist of two subdomains, Social Affect and Restricted, Repetitive, and Stereotyped Behavior, which yield one collapsed score, with two cutoffs provided: an autism cutoff score and an ASD cutoff. For Module 4, scores are obtained for two subdomains, Communication and Social, in addition to a combined subdomain score, Communication plus Social. Two cutoff scores are provided for each domain: autism and ASD. Individuals must meet all three cutoff scores in order to receive an ADOS classification of either autism or ASD.

These cutoffs differ according to which module is used. Modules 1, 2, and 3 derive a Total raw score from the Social Affect algorithm score and the Restricted, Repetitive, and Stereotyped Behaviors algorithm score. Module 1 (*n* = 2) is for those without no speech or single words, who do not use phrase speech consistently (total raw score ≥16 = autism, ≥11 = ASD). Module 2 (*n* = 1) is for those who use phrase speech but are not verbally fluent (Total raw score ≥9 = autism, ≥8 = ASD). Module 3 (*n* = 23) is for verbally fluent children (Total raw score ≥9 = autism, ≥7 = ASD). Module 4 (*n* = 30) is for verbally fluent adults and adolescents. Scoring parameters for Module 4 include the following: Communication total raw score ≥3 = autism, ≥2 = ASD; Social total raw score ≥6 = autism, ≥4 = ASD; Communication and Social total raw score ≥10 = autism, ≥7 = ASD [23].

## Figures and Tables

**Table 1:** Parent report of known medical, developmental, and family history.

	Child	Adult	Total	Family
	*n* = 24	*n* = 32	*n* = 56	
Mean age (Standard deviation)	9.58 (1.91)	19.21 (4.12)	15.1 (5.85)	-
Gender				
Female, n (percent, %)	11 (45.8)	17 (53.1)	28 (50.0)	-
Male, n (%)	13 (54.2)	15 (46.9)	28 (50.0)	-
Congenital heart defect				
n (%)	16 (66.7)	17 (53.1)	33 (58.9)	1 (1.8)
Immune deficiency				
n (%)	15 (62.5)	14 (43.8)	29 (51.8)	0 (0)
Palatal anomalies				
n (%)	11 (45.8)	19 (59.4)	30 (53.6)	0 (0)
Speech-language delay				
n (%)	17 (70.8)	15 (46.9)	32 (57.1)	8 (14.3)
Attention Deficit/Hyperactivity Disorder (ADHD)				
n (%)	11 (45.8)	7 (21.9)	18 (32.1)	12 (21.4)
Autism spectrum diagnosis (known prior to study entry)				
n (%)	0 (0)	1 (3.1)	1 (1.8)	2 (3.6)
22q11.2 deletion				
n (%)	24 (100)	32(100)	56 (100)	1 (1.8)

## Abbreviations

22q11.2DS	22q11.2 Deletion Syndrome
FISH	Fluorescent In Situ Hybridization
ASD	Autism Spectrum Disorder
ADOS	Autism Diagnostic Observation Schedule
ADI-R	Autism Diagnostic Interview-Revised
CNV	Copy Number Variation
PDD-NOS	Pervasive Developmental Disorder-Not Otherwise Specified
RDoC	Research Domain Criteria
DSM-IV	Diagnostic and Statistical Manual of Mental Disorders, Fourth edition
